# Isolated perfused working hearts provide valuable additional information during phenotypic assessment of the diabetic mouse heart

**DOI:** 10.1371/journal.pone.0204843

**Published:** 2018-10-01

**Authors:** Tina M. Pedersen, Neoma T. Boardman, Anne D. Hafstad, Ellen Aasum

**Affiliations:** Cardiovascular Research Group, Department of Medical Biology, Faculty of Health Sciences, UiT-The Arctic University of Norway, Tromsø, Norway; Temple University School of Medicine, UNITED STATES

## Abstract

Although murine models for studying the development of cardiac dysfunction in diabetes mellitus are well established, their reported cardiac phenotypes vary. These reported divergences may, in addition to the severity of different models, also be linked to the methods used for cardiac functional assessment. In the present study, we examined the functional changes using conventional transthoracic echocardiography (*in vivo*) and isolated heart perfusion techniques (*ex vivo*), in hearts from two mouse models; one with an overt type 2 diabetes (the *db/db* mouse) and one with a prediabetic state, where obesity was induced by a high-fat diet (HFD). Analysis of left ventricular function in the isolated working hearts from HFD-fed mice, suggested that these hearts develop diastolic dysfunction with preserved systolic function. Accordingly, *in vivo* examination demonstrated maintained systolic function, but we did not find parameters of diastolic function to be altered. In *db/db* mice, *ex vivo* working hearts showed both diastolic and systolic dysfunction. Although *in vivo* functional assessment revealed signs of diastolic dysfunction, the hearts did not display reduced systolic function. The contrasting results between *ex vivo* and *in vivo* function could be due to systemic changes that may sustain *in vivo* function, or a lack of sensitivity using conventional transthoracic echocardiography. Thus, this study demonstrates that the isolated perfused working heart preparation provides unique additional information related to the development of cardiomyopathy, which might otherwise go unnoticed when only using conventional echocardiographic assessment.

## Introduction

The transition to a more sedentary lifestyle and overnutrition, has led to increased incidence of obesity, hyperglycaemia, insulin resistance, dyslipidaemia, and metabolic syndrome—all known risk factors of cardiovascular disease. Consequently, cardiovascular disease is the primary cause of morbidity and mortality in diabetes patients. Diabetes also predisposes to a distinct cardiomyopathy defined as ventricular dysfunction in the absence of coronary heart disease or hypertension [1–3], which leads to the development of diastolic dysfunction prior to systolic dysfunction and finally heart failure.

A range of murine models is used to elucidate underlying mechanisms in the development of obesity and diabetes related cardiac dysfunction. Mice homozygous for the obese (Lep^*ob*^) and the diabetes (Lepr^*db*^) mutations are among the earliest characterized models of obesity-related insulin resistance and diabetes. In these monogenic models, leptin deficiency (*ob/ob* mice) or leptin receptor deficiency (*db/db* mice) lead to lack of satiety sensation, which consequently causes hyperphagia and hypoactivity. Studies using diabetic *db/db* mice have reported both development of ventricular remodelling and cardiac dysfunction [4–8], although there are studies reporting normal cardiac structure and function [9–11].

Diet-induced obese (DIO) mouse models are currently in extensive use in diabetic cardiomyopathy research as they recapitulate aspects of the metabolic syndrome associated with human obesity, and because they are comparable to commonly used genetically modified models. The reported cardiac phenotype from DIO models range from no change in cardiac function [12–15] to more severe remodelling and dysfunction [16–19]. Although, these differences may be linked to the variations between feeding protocols (nutritional composition of the diet and feeding initiation and duration) and the mouse strain used [20], there is also reason to believe that the techniques and modalities used for cardiac phenotyping influence the observed functional outcome. Today, assessment of cardiac function in mice primarily involves different *in vivo* techniques. The development of high-frequency transducers has made echocardiography a common modality for cardiac functional assessment of mouse hearts, and the parameters are mainly derived from M-mode and Doppler imaging. Although additional parameters from multi-planar images and speckle tracking echocardiography, as well as other imaging modalities (e.g. magnetic resonance imaging) have become more easily accessible, conventional echocardiography (M-mode and Doppler imaging) is still the most widespread *in vivo* modality [21].

The first reports of cardiac function in mice, published more than 25 years ago, describe cardiac function using isolated perfused hearts using the working mode [22;23] and the Langendorff mode [24]. Despite technical challenges due to the small size of the mouse heart, *ex vivo* assessment has proven valuable, not only in studying pharmacological effects, but also in the investigation of disease states and the impact of gene modification in the intact heart. It is important to emphasize that the *ex vivo* heart preparation has the advantage of facilitating elucidation of the ventricular performance under defined loading conditions and in the absence of neurohormonal influences, and may therefore provide unique additional information related to the development of dysfunction in the myocardium *per se*.

In the present study, we examined the functional changes in hearts from two mouse models of obesity/diabetes; the *db/db* mouse, a model of severe obesity and diabetes, and a DIO model induced by a high-fat diet, representing a model of obesity and prediabetes. Here, we compared *in vivo* cardiac function to that obtained *ex vivo*, using the most used imaging modality in small animal research (i.e. M-mode echocardiography and Doppler imaging) and isolated perfused heart techniques, respectively.

## Materials and methods

### Animals and diet

Mice were purchased from Charles River (Germany). Diet-induced obesity was obtained by feeding 5-6-week-old male C57BL/6J mice a high-fat diet (HFD) with 60% kcal from fat (lard), 20% kcal from carbohydrates and 20% kcal from protein (58Y1, TestDiet, UK; http://www.testdiet.com/Diets/High-Fat-DIO/index.html) for 20 weeks. Male diabetic *db/db* mice (C57BL/KsJ-lepr^*db*^/lepr^*db*^) arrived at an age of 8 weeks and were fed a regular chow diet until 12 weeks of age. We used age-matched mice fed a regular chow diet as controls. All mice were housed in a room with a constant temperature of 23°C and 55% humidity, with a 12:12-h reversed light:dark-cycle. They were given ad libitum access to water and their respective diets and were treated in accordance with the guidelines on accommodation and care of animals given by the European Convention for the Protection of Vertebrate Animals for Experimental and Other Scientific Purposes. The experiments were approved by the local representation of the National Animal Research Authority in Norway, and carried out in our laboratory at the University of Tromsø –The Arctic University of Norway.

### Blood sampling

Blood was collected from the saphenous vein (14 days prior to sacrificing the animals) after 4 hours of fasting or after a fasting/re-feeding protocol, where the mice were fasted for 3 hours, and then re-fed for 2 hours. Fasting blood glucose levels were measured using a glucometer system (FreeStyle Lite, Abbott, Australia), and insulin levels were measured in plasma from the same blood sample, using a mouse insulin ELISA kit (DRG Instruments GmbH, Germany). Plasma free fatty acids levels were analysed using commercial kits from Wako Chemicals (Neuss, Germany), in plasma collected following fasting/re-feeding.

### Echocardiography

Echocardiography was done using the VisualSonics 2100 Vevo Imaging System (Toronto, Canada) with a 550D probe (frequency of 35–40 MHz) during light isoflurane (1.5–2%) anaesthesia. The body temperature was monitored using a rectal thermometer, and maintained at approximately 37°C using a heated platform and a heat lamp.

A single operator imaged all mice. Left ventricular (LV) mass, wall thickness, end-systolic and end-diastolic diameter (LVID;s and LVID;d) and end-systolic and end-diastolic volumes (ESV and EDV) were determined from parasternal short-axis M-mode images of the midventricle at the level of papillary muscles. The LV mass was calculated from the measured cardiac borders in diastole, including the interventricular septum (IVS;d), LVID;d and the posterior wall (LVPW;d), using this formula: LV mass = (1.053*((LVID;d+LVPW;d+IVS;d)^3^-LVID;d^3^))*0.8. Functional parameters like stroke volume (SV), ejection fraction (EF) and fractional shortening (FS) were obtained from further calculations of the M-mode data. Ejection fraction was calculated as EF = (SV/EDV)*100 and fractional shortening was calculated from FS = (LVID;d-LVID;s)/LVID;d*100. The ratio between LV Volume and LV mass was used as a measure of hypertrophy. The early (E) and late/atrial (A) peak ventricular filling velocities, E/A ratio and deceleration time of early filling (DT) were obtained from transmitral flow, in the apical 4-chamber view. Early diastolic (E') and late diastolic (A') mitral annular myocardial velocity of the left ventricle septal wall was recorded from the 4-chamber view with pulsed-wave tissue Doppler. The E/E' was calculated as an index of LV filling pressure.

### Isolated heart perfusions

To evaluate the changes in heart function in the hearts independent of neurohormonal or loading conditions, we also characterised heart function *ex vivo*. After an intraperitoneal injection of pentobarbital, hearts were excised and perfused using a modified Krebs-Henseleit with added glucose (5mM) and palmitate (0.5 mM) bound to BSA (3%) [25]. Cardiac temperature was maintained at 37°C and data were obtained and analysed using LabChart 7Pro software (ADInstruments, Bella Vista, Australia).

One group of hearts was perfused in the working heart mode with an 8 mmHg preload and 50 mmHg afterload [25]. Left ventricular pressure changes were assessed using a 1.0-Fr conductance catheter (Millar Instruments, Houston, TX), inserted into the ventricle through the apex [26]. In another group, the hearts were perfused in the Langendorff mode [27], where left ventricular pressure was assessed using an intraventricular fluid-filled balloon. The volume of the balloon was adjusted so that the end-diastolic pressure was between 5–10 mmHg. To prevent build-up of fluid in the ventricle, a cannula (25 G) was inserted through the apex and into the lumen to allow drainage of fluid [27].

### Statistical analysis

The results are presented as means ± SE in tables and column bar graphs. In order to compare differences between two groups, unpaired Student’s t-tests were performed.

## Results

### Diet-induced obese mice

**Fig 1:**
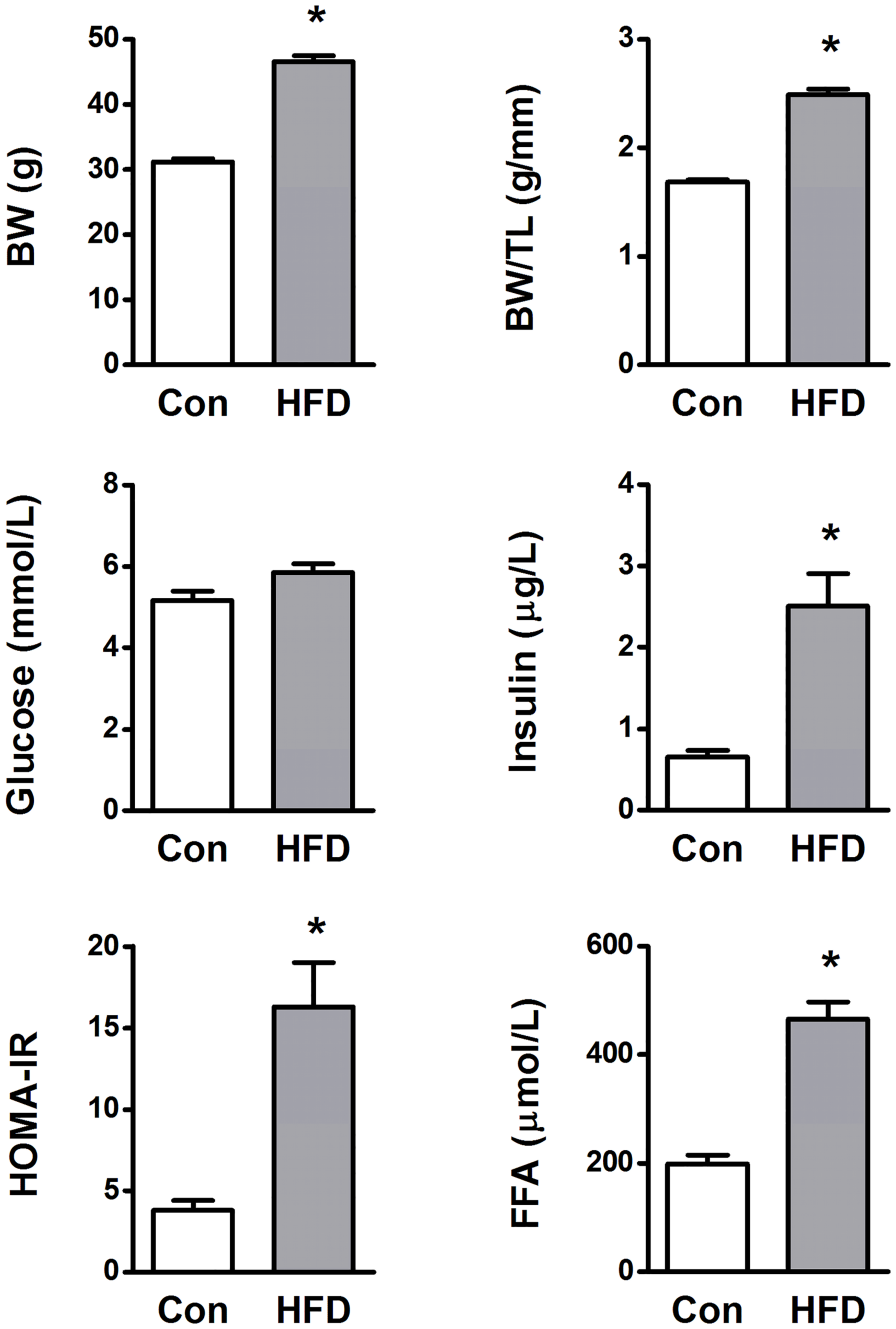
Body weight (BW), BW to tibia length (TL) ratio, blood glucose (fasted), plasma insulin (fasted), plasma free fatty acids (FFA, fasted/re-fed) and insulin resistance (HOMA-IR) from high-fat diet mice (HFD) and age-matched controls (Con). BW and tibia lengths were obtained from 25–30 mice per group, while blood samples were obtained from 13 controls and 23 HFD mice. HOMA-IR; Homeostatic Model Assessment—Insulin Resistance. * p < 0.05 vs Con.

5-6-week-old male mice were randomly divided into two groups with similar body weight (20.7 ± 0.2 g and 20.5 ± 0.2 g). Twenty weeks of feeding with the high-fat diet (HFD) resulted in obesity as indicated by higher body weight (Fig 1), and a significant increase in perirenal fat deposits (0.3 ± 0.1 vs 1.0 ± 0.1 g in controls (n = 12) and HFD (n = 13), respectively). Similar tibia lengths in the two groups of mice supports that the observation of increased weight gain in HFD mice is attributable to fat deposits and not the animal size per se (Fig 1). In this study, the HFD mice had elevated fasted blood glucose (p = 0.05, Fig 1), and HOMA-IR values (representing insulin resistance) were increased due to a marked increase in fasting insulin levels (Fig 1). In addition, HFD mice displayed a near 2.4-fold increase in free fatty acids (Fig 1).

#### *In vivo* cardiac function

Left ventricular (LV) remodelling and function *in vivo*, were assessed using transthoracic echocardiography in anesthetized mice. HFD mice did not show altered wall thickness, intraventricular diameter or LV mass (Table 1). Fractional shortening (FS) was increased in the HFD mice, due to a trend towards a decreased LV end-systolic volume (ESV, p = 0.09, Table 1). There was also a significant increase in stroke volume and ejection fraction (EF) in HFD mice, but due to a somewhat lower heart rate, cardiac output was not altered when compared to the age-matched controls (Table 1). Interestingly, the volume-mass ratio was significantly lower in HFD mice, indicating concentric hypertrophic remodelling (Table 1). Pulsed-wave tissue Doppler showed that the peak velocity of early mitral filling (E wave—depicting the velocity of blood flow in early ventricular diastolic filling), was not significantly altered. However, due to a lowered late mitral filling velocity (A wave—depicting late ventricular filling), the E/A was unexpectedly augmented in HFD hearts (Table 1). Notably though, due to the high heart rates in mice, the E and A waves are challenging to consistently measure [28], and the E/A ratio is not a reliable parameter on its own. E/E’ was unchanged, while there was a trend towards an elevated mitral valve deceleration time (p = 0.09) in the HFD mice (Table 1), indicating a mild impairment of diastolic function.

**Table 1:** Left ventricular function assessed by transthoracic echocardiography of high-fat diet-fed mice (HFD) and age-matched controls (Con).

	Con (n = 9)	HFD (n = 12)
Heart rate (BPM)	452 ± 10	439 ± 9
LVPW;d (mm)	0.79 ± 0.02	0.80 ± 0.01
LVID;d (mm)	4.1 ± 0.1	4.1 ± 0.1
LV mass (mg)	98 ± 3	107 ± 4
LVEDV (μL)	76 ± 3	74 ± 3
LVESV (μL)	36 ± 3	29 ± 2
SV (μL)	41 ± 1	45 ± 2 *
EF (%)	54 ± 2	61 ± 2 *
FS (%)	28 ± 1	32 ± 1 *
Volume/LV mass (μL/mg)	0.78 ± 0.02	0.70 ± 0.02 *
E/A	1.4 ± 0.1	1.7 ± 0.1 *
E/E'	32 ± 2	30 ± 1
DT (ms)	25 ± 1	27 ± 1

Data are means ± SE. LVPW;d and LVID;d, left ventricular (LV) posterior wall thickness and internal diameter in diastole, respectively; EDV and ESV, end-diastolic and end-systolic volumes; SV, stroke volume; EF, ejection fraction; FS, fractional shortening; E/A; ratio of velocities of early to late ventricular filling, E/E’; ratio of velocity of early ventricular filling to early diastolic mitral annular velocity, DT; deceleration time.

*p < 0.05 vs Con.

#### *Ex vivo* cardiac function

**Fig 2:**
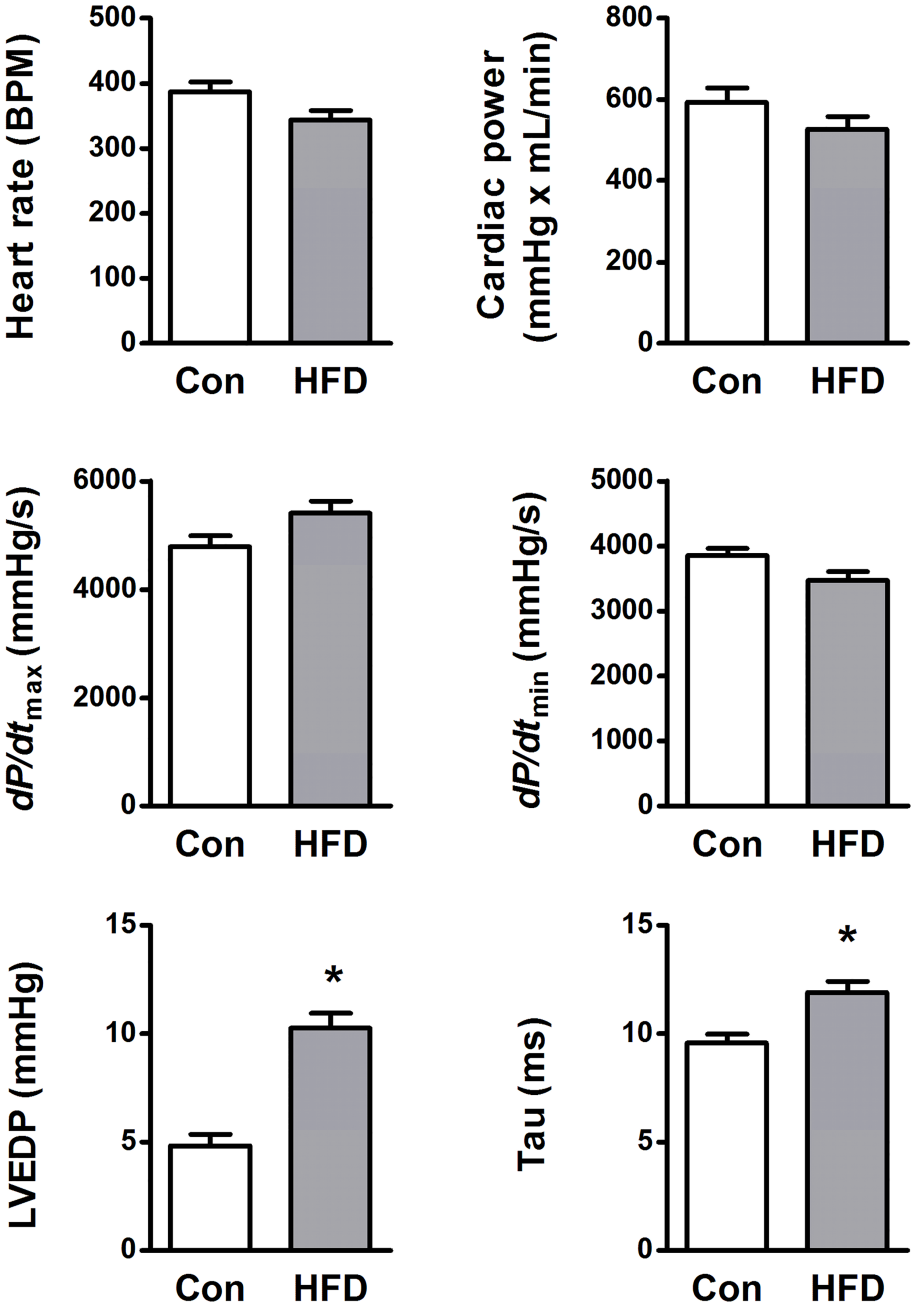
Left ventricular function assessed in isolated perfused working hearts from high-fat diet fed mice (HFD, n = 6) and age-matched controls (Con, n = 8). *dP/dt*_max_ and *dP/dt*_min_; maximum positive and negative first-time derivative of left ventricular (LV) pressure respectively, left ventricular end-diastolic pressure (LVEDP), Tau; LV relaxation time constant calculated by the Weiss method. * p < 0.05 vs Con.

Isolated perfused working hearts from HFD mice showed a slight but not significant decrease in intrinsic heart rate (p = 0.06, Fig 2). HFD hearts also displayed elevated LV end-diastolic pressure (LVEDP) and impaired relaxation as indicated by a decreased *dP/dt*_min_ (p = 0.06) and increased Tau (the relaxation time constant) (Fig 2). On the other hand, HFD hearts displayed no change in *dP/dt*_max_ or cardiac power (the product between cardiac output and LV developed pressure, Fig 2). Developed pressure (53.0 ± 1.6 vs 52.7 ± 0.9 mmHg in controls (n = 6) and HFD (n = 8), respectively) and cardiac output (11.3 ± 0.6 vs 10.8 ± 1.1 mL/min in controls (n = 6) and HFD (n = 8), respectively) were not different from controls either.

The Langendorff perfusion mode has become the most commonly used perfusion mode for evaluating *ex vivo* function in mouse hearts, as it less technically challenging when compared to the working mode. Due to the small size of these hearts, the LV balloon has to be adjusted after it has been inserted into the LV, and this is generally done so that the LVEDP is between 5–10 mmHg. In this study, the mean values of the control and HFD hearts were 6.9 ± 0.7 and 8.6 ± 0.9 mmHg, respectively. In this perfusion mode, we did not find an increase in *dP/dt*_min_, which would have indicated impaired relaxation (Table 2). Additionally, none of the parameters of systolic function (maximal systolic pressure, LV developed pressure, *dP/dt*_max_ or the rate-pressure-product) were altered in HFD hearts (Table 2).

**Table 2:** Left ventricular function assessed in isolated Langendorff perfused hearts from high-fat diet-fed mice (HFD) and age-matched controls (Con).

	Con (n = 12)	HFD (n = 12)
Heart rate (BPM)	318 ± 11	307 ± 14
Coronary flow (mL/min)	3.2 ± 0.2	3.4 ± 0.4
Developed pressure (mmHg)	139 ± 8	139 ± 14
*dP/dt*_max_ (mmHg/sec)	5282 ± 367	5623 ± 552
*dP/dt*_min_ (mmHg/sec)	-3610 ± 119	-3654 ± 292
RPP (mmHg x BPM)	44579 ± 3403	42238 ± 4366

Data are means ± SE. *dP/dt*_max_ and *dP/dt*_min_; maximum positive and negative first-time derivative of left ventricular (LV) pressure respectively. RPP; rate-pressure-product (the product of developed pressure and heart rate).

### *db/db* mice

**Fig 3:**
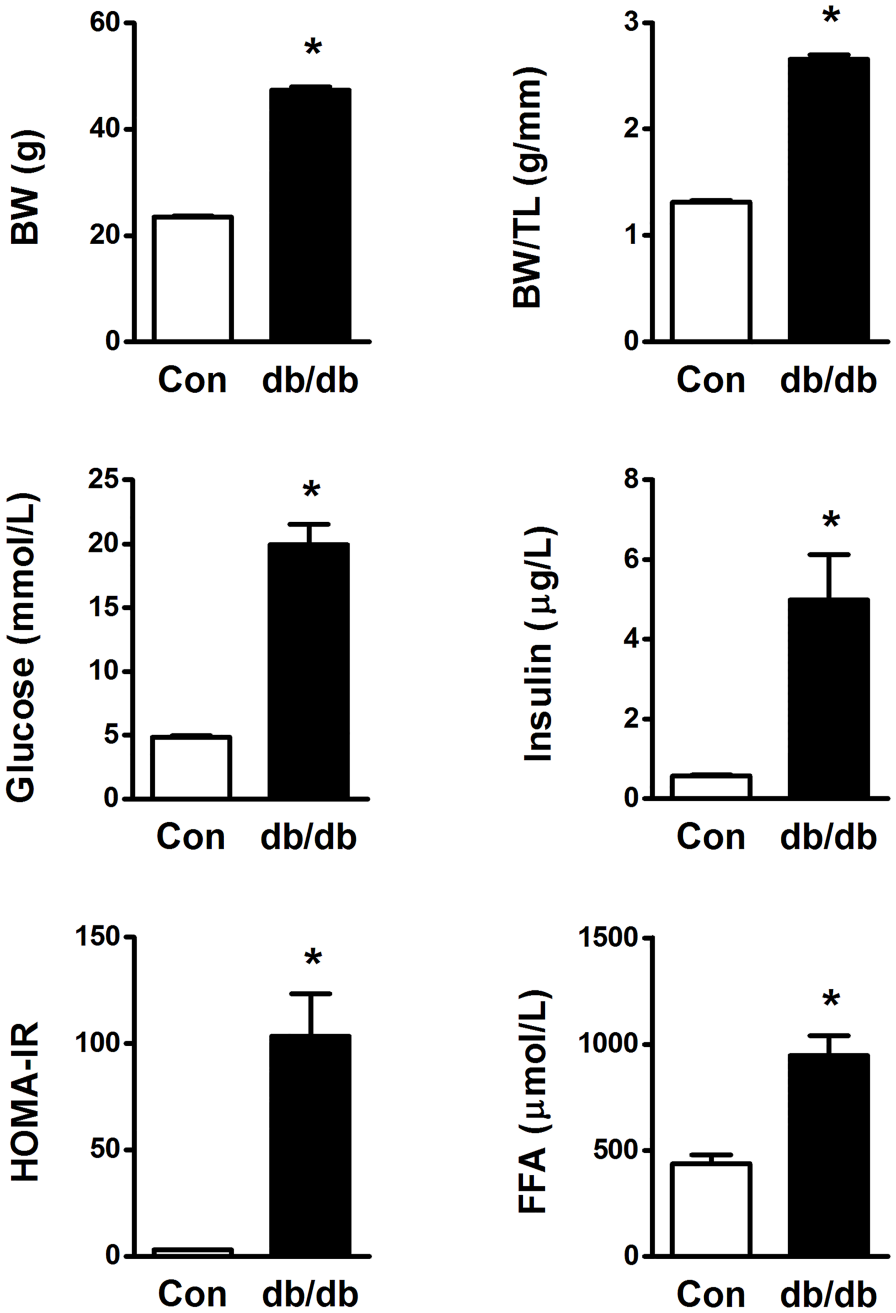
Body weight (BW), BW to tibia length (TL) ratio, blood glucose (fasted), plasma insulin (fasted), plasma free fatty acids (FFA, fasted/re-fed) and insulin resistance from *db/db* mice and age-matched controls (Con). BW and tibia lengths were obtained from 25–28 mice per group, while blood samples were obtained from 11 controls and 6 *db/db* mice. HOMA-IR; Homeostatic Model Assessment—Insulin Resistance. * p < 0.05 vs Con.

12-week-old *db/db* mice had twice the body weight of the age-matched lean mice (Fig 3). The *db/db* animals also displayed a substantial elevation in HOMA-IR level due to a considerable increase in both fasting insulin and glucose levels. We also found plasma free fatty acids levels to be significantly increased in the *db/db* mice (Fig 3).

#### *In vivo* cardiac function

Echocardiographic examination revealed elevated LV masses in *db/db* mice (Table 3), which is in contrast to previous data obtained by weighing these hearts [25;29]. This discrepancy may be related to potential differences in myocardial density, which is not included in the LV mass calculation. There was no change in heart rate between the two groups, but the *db/db* hearts showed increased LV diastolic diameter and end-diastolic volume (EDV, Table 3). As systolic diameter and ESV was unchanged, stroke volume and cardiac output were significantly increased (Table 3). These changes were also associated with a significant increase in EF and FS (Table 3). Doppler measurements showed that the E wave was decreased in *db/db* hearts (p = 0.003), but since the A wave was also decreased (p = 0.04), the E/A remained unchanged (Table 3). Regardless of the unchanged ratio, the lowered filling velocities might portray increased resistance in the ventricle, and as the *db/db* hearts also showed a marked increase in the E/E’ and mitral deceleration time (Table 3), this proves that these hearts have decreased ventricular compliance and increased myocardial stiffness.

**Table 3:** Left ventricular function assessed by transthoracic echocardiography of *db/db* mice and age-matched controls (Con).

	Con (n = 8)	*db/db* (n = 10)
Heart rate (BPM)	454 ± 10	464 ± 10
LVPW;d (mm)	0.76 ± 0.03	0.81 ± 0.02
LVID;d (mm)	3.83 ± 0.05	4.00 ± 0.04 *
LV mass (mg)	86 ± 4	100 ± 3 *
LVEDV (μL)	63 ± 2	70 ± 2 *
LVESV (μL)	23 ± 2	21 ± 1
SV (μL)	41 ± 2	49 ± 2 *
EF (%)	64 ± 2	70 ± 1 *
FS (%)	35 ± 2	39 ± 1 *
Volume/LV mass (μL/mg)	0.74 ± 0.02	0.71 ± 0.03
E/A	1.6 ± 0.1	1.6 ± 0.1
E/E'	30 ± 1	41 ± 1 *
DT (ms)	26 ± 1	31 ± 2 *

Data are means ± SE. LVPW;d and LVID;d, left ventricular (LV) posterior wall thickness and internal diameter in diastole, respectively; EDV and ESV, end-diastolic and end-systolic volumes; SV, stroke volume; EF, ejection fraction; FS, fractional shortening; E/A; ratio of velocities of early to late ventricular filling, E/E’; ratio of velocity of early ventricular filling to early diastolic mitral annular velocity, DT; deceleration time.

*p < 0.05 vs Con.

#### *Ex vivo* cardiac function

**Fig 4:**
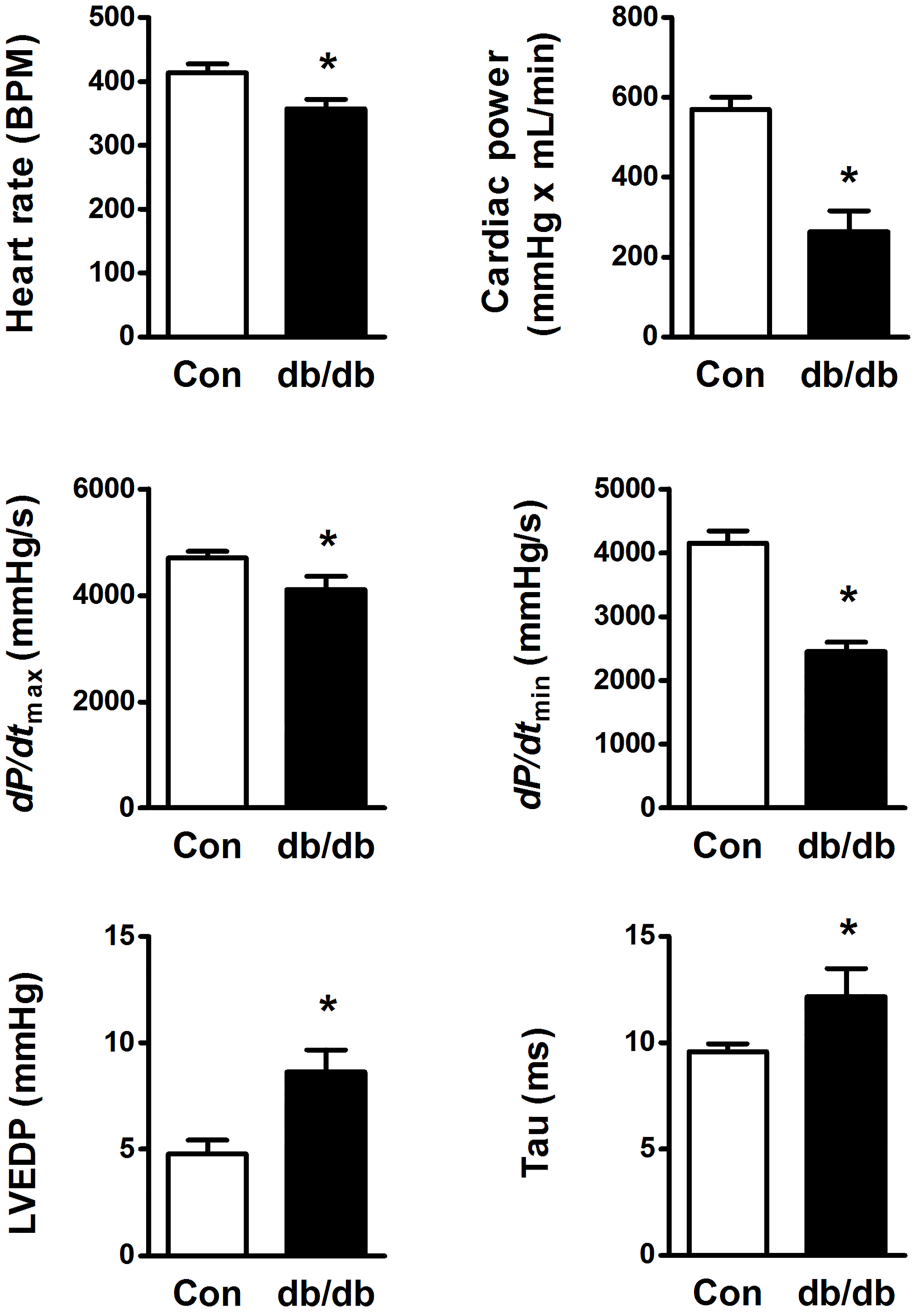
Left ventricular function assessed in isolated perfused working hearts from *db/db* mice (n = 10) and age-matched controls (Con, n = 7). *dP/dt*_max_ and *dP/dt*_min_; maximum positive and negative first-time derivative of left ventricular (LV) pressure respectively, left ventricular end-diastolic pressure (LVEDP), Tau; LV relaxation time constant calculated by the Weiss method. * p < 0.05 vs Con.

In the isolated working mode, the *db/db* hearts showed a diastolic dysfunction as indicated by increased LVEDP, lowered *dP/dt*_min_ and increased Tau (Fig 4). In addition, a combination of reduced stroke volume (23.8 ± 1.3 vs 14.6 ± 2.7 μL in controls (n = 10) and *db/db* (n = 7), respectively) and lowered heart rate (Fig 4), led to a marked decrease in cardiac output (9.8 ± 0.4 vs 5.1 ± 0.8 mL/min in controls (n = 10) and *db/db* (n = 7), respectively). These hearts also showed decreased cardiac power (Fig 4) and finally, despite the lower HR, a significantly lower LV developed pressure (58.2 ± 1.4 vs 50.7 ± 2.4 mmHg in controls (n = 10) and *db/db* (n = 7), respectively) and *dP/dt*_max_ (Fig 4), which further supports a decreased systolic function in these hearts. It is, however, important to appreciate that the impaired cardiac function in these hearts could also be related to the HR.

In Langendorff perfused hearts, the volume of the balloon was adjusted so that the LVEDP mean values were 6.2 ± 0.6 and 6.7 ± 0.2 mmHg in the control and *db/db* hearts, respectively. We did not find *dP/dt*_min_ to be altered in the *db/db* hearts, and neither were parameters of systolic function (LV developed pressure, rate-pressure-product and *dP/dt*_max_) (Table 4).

**Table 4:** Left ventricular function assessed in isolated Langendorff perfused hearts from *db/db* mice and age-matched controls (Con).

	Con (n = 8)	*db/db* (n = 10)
Heart rate (BPM)	292 ± 26	269 ± 22
Coronary flow (mL/min)	2.7 ± 0.2	2.3 ± 0.2
Developed pressure (mmHg)	133 ± 13	123 ± 8
*dP/dt*_max_ (mmHg/sec)	4793 ± 672	4844 ± 446
*dP/dt*_min_ (mmHg/sec)	-3362 ± 335	-3271 ± 234
RPP (mmHg x BPM)	36778 ± 4954	30683 ± 2690

Data are means ± SE. *dP/dt*_max_ and *dP/dt*_min_; maximum positive and negative first-time derivative of left ventricular (LV) pressure respectively. RPP; rate-pressure-product (the product of developed pressure and heart rate).

## Discussion

The present study demonstrates differences between the *ex vivo* and *in vivo* cardiac functional phenotype within the same murine models of diabetes. While isolated perfused working hearts from diabetic mice showed clear signs of dysfunction, conventional transthoracic echocardiography did not reveal this to the same extent. Although lack of *in vivo* cardiac dysfunction may be due to a low sensitivity of the M-Mode and Doppler assessment, it may also relate to systemic factors (such as altered neurohormonal status and/or changes in pre- and afterload) which might mask subtle functional changes *in vivo*. This study therefore shows how *ex vivo* examination can add valuable information when describing the progression of cardiomyopathy.

### The cardiac phenotype in diet-induced obese mice

In accordance with previous studies, mice fed a high-fat diet (HFD) for 20 weeks were in a prediabetic state with obesity, hyperlipidaemia and insulin resistance, but without hyperglycaemia. In most studies, long-term high-fat feeding will increase left ventricular (LV) mass and induce LV concentric hypertrophy [17;30–34]. The reported incidence of systolic and diastolic dysfunction is, however, inconsistent. Systolic dysfunction has been reported following feeding regimes that varied from 4–6 [33;35] to 15–16 weeks [19;32;36], while the present study and other studies report no evidence of systolic dysfunction after 20–24 weeks [15;27;31;37;38]. In general, diastolic dysfunction is considered to advance prior to the development of systolic dysfunction, and although some studies [17;27;34] support this by showing impaired diastolic dysfunction without systolic dysfunction, the duration of HFD feeding required to evoke diastolic impairments remains unclear. Additionally, as the functional modalities/techniques have different sensitivities, lack of reported diastolic dysfunction by one modality/technique, does not necessarily exclude its occurrence. In accordance with this, a recent study by Schnelle and colleagues [28] shows how combining several ultrasound-based techniques of diastolic function (including additional views and speckle tracking echocardiography) will not only improve the detection of diastolic dysfunction in murine models, but also reveal the underlying pathophysiology for this dysfunction.

The present study showed that while the echocardiographic parameters (obtained by M-mode and Doppler) did not demonstrate diastolic dysfunction, *ex vivo* assessment (using perfused working hearts) confirmed previous reports of diastolic dysfunction [16;27]. This shows how *ex vivo* assessment of function using the working heart mode represents a sensitive and additional method for evaluation of diastolic dysfunction.

### The cardiac phenotype in *db/db* mice

The *db/db* mouse represents a type 2 diabetic mouse model, as indicated by obesity, insulin resistance, hyperglycaemia and hyperlipidaemia. These hearts show an age-dependent increase in LV mass and wall thickness [5;11;39;40], but the literature is not consistent with regard to the development of cardiac dysfunction. Semeniuk and colleagues [4] were the first to describe echocardiographic examination of the *db/db* mice in 2002, where they reported reduced fractional shortening (FS) and E/A in 12 but not 5-week-old mice. Consistent with this, studies from our laboratory later reported that *ex vivo* working hearts from 12, but not 5-6-week-old mice, showed severe dysfunction [25;41–45]. We were therefore surprised to find that echocardiographic examination in the present study only revealed a mild diastolic impairment with no (or even improved) systolic dysfunction. The literature on echocardiographic examination of *db/db* mice (14–18 weeks of age), have reported both reduced [8], unaltered [9;11], and increased systolic function [10]. Although this inconsistency could be due to differences in the severity of the diabetic phenotype caused by using different background strains in these mice [46], it may also reflect the lack of sensitivity of conventional echocardiography.

Accordingly, Yue and colleagues [5] reported normal function in young (5-week-old) mice, but reduced FS and increased end-diastolic volume at an age of 9–13 weeks when using MRI. Interestingly, despite decreased FS, they found a slight (although not significant) increase in cardiac output at all ages. Again, the literature is inconsistent, as other studies (using the same modality) have not reported the same changes [6;47]. It should be noted that Stuckey *et al*. reported impairments in contractility and diastolic function in 12-week-old mice when analysing data from high-temporal resolution MRI, despite finding no functional changes when using standard MRI [6]. Interestingly, maintained systolic function in 12-week-old mice was supported both by a recent study by Li et al. [48] using both STE and conventional echocardiography. In this study only STE revealed a dysfunction when these mice were 16 weeks old [48], which suggests that conventional echocardiography lacks the sensitivity to capture subtle variations in left LV performance.

The very first functional assessment of the *db/db* hearts was not *in vivo* but performed using the isolated perfused working heart. In this study, Belke and colleagues measured intraventricular pressure (using a fluid-filled catheter) and demonstrated a higher end-diastolic pressure, as well as reduced cardiac output and cardiac power in hearts from 10-14-week-old mice [49]. Impaired *ex vivo* systolic function was later confirmed in 12-week-old mice [25;41;50]. In accordance with previous studies [29;44], the present study confirmed both diastolic and systolic dysfunction, using intraventricular pressure-volume recordings, which allow more accurate assessment of a range of load-dependent and -independent functional parameters.

It should be noted that when these hearts were perfused in the Langendorff mode, there were no detectable changes in parameters of LV function at neither 12 (present study) nor 24 weeks [47]. Thus, although the Langendorff mode is a suitable *ex vivo* mode for vascular reactivity studies and acute studies of changes in contractility following drug or ischemic damage, the present study reinforces that this mode is less suitable for detailed functional assessment, particularly in models of diastolic dysfunction.

### The cardiac phenotype obtained *in vivo* vs *ex vivo*

The functional capacity of the *in vivo* heart is determined by the endogenous working capacity of the myocardium, coronary perfusion and changes in systemic factors known to influence the myocardial function, such as loading conditions and neurohormonal status. Thus, the functional phenotype of the diabetic heart may be altered due to pathological alterations in the myocardium itself and/or systemic changes representing compensatory or pathophysiological alterations.

In the present study, as well as in previous reports, there seems to be a discrepancy between the *in vivo* and *ex vivo* function in *db/db* hearts, when comparing parameters obtained by conventional echocardiography and the isolated perfused working hearts. As the mechanical function of the hearts *ex vivo* is examined under identical loading and neurohormonal conditions, it could be argued that favourable load-dependent and/or load-independent changes may have contributed to improve *in vivo* function. Haemodynamic measurements using LV *in vivo* catheterization in *db/db* hearts have supported this notion, first by showing impaired *load-independent* parameters of diastolic dysfunction (increased slope of the end-diastolic pressure-volume relationship) [5;51–53] and contractility (diminished preload recruitable stroke-work index and end-systolic elastance) [5;51]. On the other hand, *load-dependent* parameters of systolic function (*dP/dt*_max_ and cardiac output) were reported to be elevated [50;51], strongly supporting changes in the pre- and/or after-load. The increased LVEDP [50–52] and decreased arterial elastance in these mice [51;53] (both *load-dependent* parameters), have been argued to signify increased preload and decreased afterload, respectively [51], further supporting the notion of compensated haemodynamic conditions in *db/db* hearts.

Neurohormonal changes (as a compensatory mechanism) may also affect cardiac function *in vivo*. Discrepancy between the *in vivo* and *ex vivo* heart function has also been reported after a genetic impairment of SERCA2 function in the heart. While isolated cardiomyocytes from these hearts showed impaired cell shortening and reduced Ca^2+^-transient amplitude [54], and isolated perfused working hearts displayed severe heart failure [55], echocardiographic examination showed a near normal *in vivo* function [54]. In support of this, Land et al. [56] demonstrated, using computational whole-organ simulation, the important role of the compensatory systemic changes to maintain *in vivo* function. They showed that the model would only be able to match the reported *in vivo* function if they included the effect of β-adrenergic stimulus i.e. the enhanced Ca^2+^-transient amplitudes, and increased venous return with its subsequent Frank-Starling effect [56]. The fact that cardiomyocytes from the *db/db* hearts also show impaired SERCA2 function and an accompanying decrease in Ca^2+^-transient amplitude and cell shortening [57;58], further supports that compensatory systemic changes can contribute to sustain *in vivo* cardiac function in these mice.

As the β-adrenergic drive also influences heart rate (HR), a lower HR in *db/db* hearts *ex vivo* [25;44;50] but not *in vivo* [39;48;51], supports that the neurohormonal status is altered in *db/db* mice. Although we cannot fully exclude that the reduced *ex vivo* HR in *db/db* hearts could have contributed to the reduced function, decreased contractility and impaired relaxation have also been reported in studies where HR was not significantly different in isolated perfused *db/db* hearts [29;45].

### Limitations

The present study, cannot fully exclude the presence of *in vivo* systolic or diastolic dysfunction. Here we used the M-mode and Doppler images, as these are the most widely used images, and are regarded as suitable approaches for assessment of heart disease where the structural remodelling develop in a uniform manner [21]. It should be noted, however, that a recently published guideline for measuring cardiac physiology in mice [21] recommends that detection of subtle systolic changes requires the use of other imaging modalities or techniques, to give a comprehensive and full description of cardiac function.

Oxygen delivery/supply can limit the function of an *ex vivo* heart, as demonstrated by altered function in rabbit hearts perfused with a buffer that had increased oxygen carrying capacity [59]. To what extent this also relates to smaller hearts (such as the mouse heart) is not known, and whether a resulting decrease in myoglobin oxygen saturation will have an impact on oxidative phosphorylation, remains unresolved [59]. In addition, it is not clear whether the function of the diabetic heart is more sensitive to mild hypoxia, and thus we do not know to what extent this may have influenced the systolic dysfunction observed *ex vivo*, but not *in vivo*, in diabetic hearts.

## Conclusion

Although continued advances in *in vivo* imaging will provide access to new and more sensitive modalities for cardiac phenotyping, this study demonstrates that the isolated heart preparation remains a valuable tool for assessment of the myocardial function *per se*, and by that may bridge *in vitro* assays and *in vivo* approaches.
